# Gut microbiota affects the progression of colorectal cancer under the intervention of exercise

**DOI:** 10.3389/fmicb.2026.1728541

**Published:** 2026-02-05

**Authors:** Linlin Tao, Huan Zhou, Wenjiao Shao, Dongmei Liu, Yingwen Ruan, Mingwei Chen

**Affiliations:** 1 Department of Human Anatomy, Harbin Medical University, Harbin, China; 2 Department of Radiology, Renmin Hospital of Wuhan University, Wuhan, China; 3 Department of Gynecological Radiotherapy, Harbin Medical University Cancer Hospital, Harbin, China

**Keywords:** colorectal cancer, exercise intervention, gut microbiota, inflammation, intestinal barrier

## Abstract

Colorectal cancer (CRC) ranks as the third most common cancer globally, accounting for 10% of cases, and is the second leading cause of cancer-related death. Exercise can maintain/restore the body’s internal balance at multiple levels to prevent pathologies, although its mechanism is complex and involves multiple systems. While physical activity improves colon cancer patients’ quality of life and reduces discomfort, how it works remains unclear. To explore this, we constructed a rat CRC model. We performed 16S rRNA and nontargeted metabolomics sequencing and analyzed the results to screen distinct intestinal microbes and their metabolites. To clarify the links among these microbes/metabolites, intestinal barrier function, and inflammatory markers, we used qRT–PCR, immunohistochemistry, and ELISA to verify the effects of exercise intervention on intestinal barrier function and inflammatory factor expression. We also checked the expression of the PCNA gene (a proliferation index) to assess cell proliferation. *In vivo*, different exercise interventions hindered DMH-induced colon cancer progression in rats and reshaped the gut microbiota. They increase the number of beneficial bacteria, such as *Bifidobacterium*, and reduce the number of harmful bacteria, such as *Escherichia-Shigella*. Compared with the Model group, exercise restored intestinal barrier function, promoted anti-inflammatory factor expression, and suppressed proinflammatory factor expression by regulating the gut flora. Overall, exercise intervention curbs CRC development by modulating gut flora–metabolite interactions, restoring the intestinal barrier, and adjusting inflammatory expression. This study confirms the inhibitory effect of exercise on CRC progression. These findings show that different intensities of exercise can impact CRC intestinal barrier function and inflammatory factor levels by balancing the gut flora and metabolites, suggesting a new CRC prevention/treatment approach.

## 1 Introduction

CRC is the third most common cancer and second leading cause of cancer death globally (Kazemi et al., 2023). Extensive research (Hojman et al., 2018; Telles et al., 2022; Schon and Weiskirchen, 2016; Zhao et al., 2019; Singh et al., 2020) confirms that aerobic exercise intervention prevents disease onset, inhibits disease progression, and improves patient prognosis, underscoring its key role in disease prevention and treatment.

An imbalance in the gut microbiota is closely linked to the development of CRC (Chattopadhyay et al., 2021). The gut microbiota colonizes (Wong and Yu, 2019) the colorectal epithelium, participates in dietary metabolism, and plays a crucial role in maintaining bodily balance. Dysbiosis is characterized by the proliferation of pathogens (Chow and Mazmanian, 2010), a reduction in commensals (Buffie et al., 2014), and a decrease in diversity (Levy et al., 2017). *Fusobacterium nucleatum* and other bacteria can promote CRC progression through different signaling pathways (Tomkovich et al., 2017; Boleij et al., 2015). Pathogenic microorganisms (Cao et al., 2022; Goodwin et al., 2011) can also produce genotoxins or cause deoxyribonucleic acid (DNA) damage via indirect mechanisms, thereby promoting tumor formation. Therefore, elucidating the molecular mechanisms underlying the imbalance between pathogenic bacteria and antitumor probiotics during gut dysbiosis could open new avenues for the prevention and treatment of CRC.

The structure of the gut microbiota (Avuthu et al., 2022) is complex and dynamic. It can alter host metabolism through various pathways, thereby affecting the development of colorectal cancer. Short-chain fatty acids (SCFAs) are a type of metabolite that is influenced by the gut microbiota and is closely associated with CRC (Wang et al., 2011). The levels of SCFAs in the feces of CRC patients are significantly decreased, and the root cause lies in the reduction in the number of microorganisms, such as the *Lachnospira*, that produce SCFAs. Specifically, butyrate, an SCFA, is deeply involved in key cellular activities, such as DNA methylation and cell cycle regulation, and SCFAs as a whole are also associated with programmed death-ligand 1 (PD-L1) therapy (Mirzaei et al., 2021). Another metabolomics study (Bai et al., 2022) also provided new evidence that, in the colon of mice exposed to smoke, the bile acid metabolite taurodeoxycholic acid (TDCA) increases significantly. This phenomenon is closely related to *Eggerthella lenta*, and the mitogen-activated protein kinase (MAPK)/extracellular signal-regulated kinase (ERK) signaling pathway is also involved, as it is associated with changes in TDCA levels and damage to the intestinal barrier. In addition, a high-fat diet (Enos et al., 2016) can also change the structure of the gut microbiota in mice, leading to an increase in pathogenic bacteria, a decrease in probiotics, and an increase in the related metabolite glycerophospholipids, ultimately resulting in damage to intestinal barrier function.

**Figure 1:**
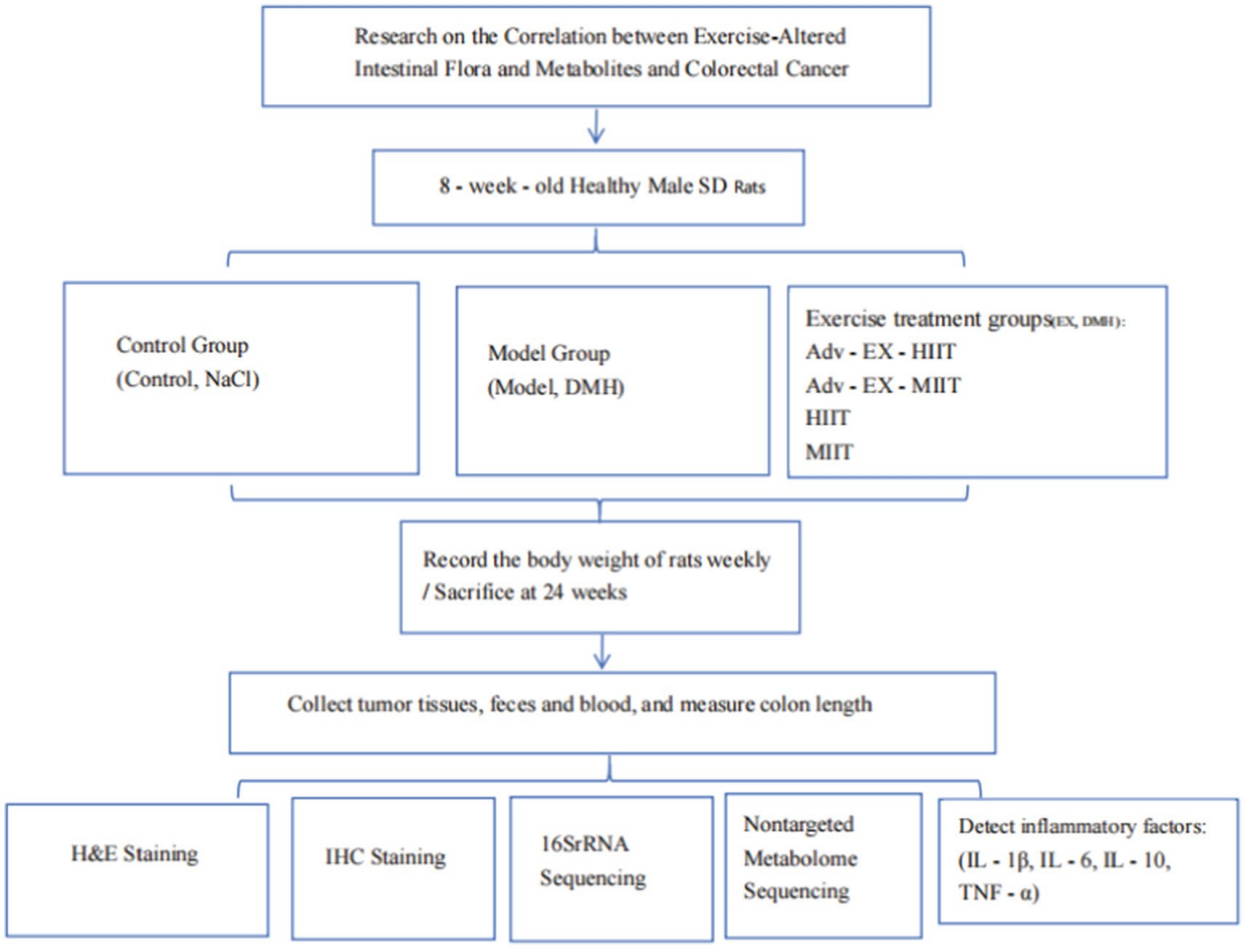
Technology road map. Adv-EX-HIIT, Pre-aerobic exercise with high-intensity interval training; Adv-EX-MIIT, Pre-aerobic exercise with moderate-intensity interval training; HIIT, High-intensity interval training; MIIT, Moderate-intensity interval training.

In this study, we used aerobic exercise interventions at different intensities and stages. Our goals were to determine whether CRC alters the intestinal microbiota composition, understand the role of these changes in CRC, and uncover the molecular mechanisms of CRC progression. We aimed to identify better CRC prevention and treatment strategies. The experimental roadmap of this study is shown in Figure 1.

## 2 Materials and methods

### 2.1 Experimental animals

Thirty-six 8-week-old male Sprague–Dawley (SD) rats were purchased from the Laboratory Animal Center of the Second Affiliated Hospital of Harbin Medical University. All breeding conditions were carried out in accordance with standard specifications. After a one-week adaptation period, the rats were randomly divided into six groups: the control group and the DMH group. Adv-Ex-HIIT group (high-intensity intermittent exercise intervention was performed in advance before the DMH was injected), Adv-Ex-MIIT group (mid-intensity intermittent exercise intervention was performed in advance before the DMH was injected), HIIT group (high-intensity intermittent exercise intervention was performed in progress while the DMH was injected), and MIIT group (mid-intensity intermittent exercise intervention was performed in progress while the DMH was injected). The weights were recorded every week, and after 24 consecutive weeks of feeding, the blood, colon, and fecal tissues were removed, and the samples were stored in a −80° refrigerator for later experiments.

### 2.2 Sports intervention program

A simplified schematic diagram of the exercise protocol is provided in Supplementary Figure S1.

Aerobic exercise programme: After 1 week of adaptation, the exercise group was forced to run on the treadmill. The running exercise training included a 2-week adaptive training phase, followed by a sports training phase (with 2 days off per week).

For the Adv-Ex-HIIT group, the first day starts at 8 m/min, lasts for 15 min, at 10 m/min on the second day, lasts for 30 min, at 10 m/min on the third day, lasts for 30 min, at 15 m/min on the fourth day, lasts for 45 min, at 15 meters/min on the fifth day, lasts for 45 min, at 20 meters/min on the sixth day, lasts for 45 min, at 20 meters/min on the seventh day, lasts 50 min, at 22 meters/min on the eighth day, lasts 60 min, at 22 meters/min on the ninth day, lasts 60 min, and lasts 60 min, at 25 meters/min on the last tenth day, lasting 66 min. The Adv-Ex-MIIT group, similar to the Adv-Ex-HIIT group, lasts for the first 7 days, lasts 20 meters/min on the eighth day, lasts 60 min, and lasts 20 meters/min on the ninth day, lasting 66 min, and lasts 66 min, and lasts 66 min. After the speed and duration of exercise were determined, 1 month of exercise training was performed in advance for both the HIIT group and the MIIT group, and exercise training was continued until the end of modeling. After that, the HIIT group and the MIIT group also conducted 2 weeks of adaptive training, with the acceleration cycle and time being the same as those of the Adv-Ex-HIIT group and the Adv-Ex-MIIT group. After 2 weeks, the exercise training phase began until the modeling ended.

In the following exercise training stage, the rats in the Adv-Ex-HIIT group and Adv-Ex-MIIT group were subjected to two intensities of advanced exercise intervention and were running on a 0-degree inclined treadmill. The two groups were trained for 66 min at speeds of 25 m/min and 20 m/min each day, 5 days a week, for 24 weeks. The HIIT group and the MIIT group were in the same mode as the Adv-Ex-HIIT group and the Adv-Ex-MIIT group for 20 weeks.

Intermittent exercise training mode: Adv-Ex-HIIT group and HIIT group (5-min warm-up speed: 8 meters/min; 8 interval training modes: 25 meters/min speed lasting for 5 min, 2-min rest mode; 5-min cooling speed: 8 meters/min). The Adv-Ex-MIIT group and MIIT group (5-min warm-up speed: 8 meters/min; 8 interval training modes: 20 meters/min speed lasting for 5 min, 2-min rest mode; 5-min cooling speed: 8 meters/min) (Yang et al., 2022; Wang et al., 2020; Shamsipour et al., 2021; Guo et al., 2022; Reljic et al., 2021).

### 2.3 Immunohistochemistry (IHC)

Paraffin sections were incubated at 60 °C for 20–30 min, dewaxed in xylene, rehydrated in graded ethanol, and rinsed with phosphate-buffered saline (PBS). Antigen retrieval was performed with sodium citrate in a microwave oven, followed by blocking peroxidase activity with 3% hydrogen peroxide and serum. The samples were incubated with primary antibody at 4 °C overnight, rewarmed at 37 °C the next day, washed, and incubated with secondary antibody for 1 h, followed by another wash. After 3,3′**-**diaminobenzidine (DAB) staining and counterstaining with hematoxylin, the samples were washed, dehydrated, cleared, mounted, and then imaged under an upright microscope. The antibodies used in this experiment are shown in Supplementary Table S1.

### 2.4 Quantitative real-time PCR (qRT–PCR)

The primers used in the experiment were designed via Primer 3 online software and synthesized by Sangon Bioengineering Co., Ltd. The specific sequences of the primers used are provided in Supplementary Table S2.

We extracted total ribonucleic acid (RNA) from rat tissue via a standard protocol and performed reverse transcription and qRT–PCR. The specific components and procedures are listed in Supplementary Tables S3–S5.

### 2.5 Hematoxylin and eosin (H&E) tissue staining

Paraffin sections were heated at 60 °C for 20–30 min, dewaxed in xylene (I and II, 15 min each), rehydrated in graded ethanol (100%, 90%, 80%, 70%) for 5 min each, and rinsed with PBS 3 times for 3 min each. Nuclei were stained with hematoxylin for 3–5 min (adjusted under a microscope), rinsed with tap water for 10 min to turn blue, and the cytoplasm was stained with eosin for 1–3 min. The samples were dehydrated in graded ethanol (75%, 95%, 100%) for 1 min each, cleared in xylene (I and II, 5 min each), mounted with neutral balsam, air-dried, and then examined and photographed under a microscope.

### 2.6 Enzyme-linked immunosorbent assay

The ELISA was meticulously carried out in strict accordance with the detailed protocols furnished within the specific kit. For comprehensive information regarding the kit, including its brand and manufacturer details, please refer to Supplementary Table S6.

### 2.7 16S ribosomal ribonucleic acid sequencing

Total RNA from diverse microbial groups was extracted via the cetyltrimethylammonium bromide (CTAB) method, with extraction efficiency assessed via agar gel electrophoresis and RNA content determined via ultraviolet spectroscopy. The PCR products were purified via AMPureXT beads, quantified via a Qubit, identified via 2% agarose gel electrophoresis, and recovered with the same beads. The purified products were evaluated via an Agilent 2100 Bioanalyzer and an Illumina Kapa kit, with libraries ≥2 nM selected. Qualified libraries (with unique indices) were pooled proportionally, denatured to single strands with sodium hydroxide, and sequenced on a NovaSeq 6000 (2 × 250 bp paired-end) using a 500-cycle SP reaction box as a control. The data were subjected to demultiplexing, splicing, filtering, diversity analysis, species annotation, differential analysis, and advanced analyses. The specific experimental procedures are provided in Supplementary Tables S7–S9.

### 2.8 Nontargeted metabolite sequencing

A Thermo UltiMate 3000 high-performance liquid chromatography system was coupled with an ACQUITY UPLC BEH C18 column (100 mm × 2.1 mm, 1.8 μm), with a column temperature of 35 °C and a flow rate of 0.4 mL/min. The mobile phases were 0.1% formic acid (A) and 0.1% formic acid in acetonitrile (B). Metabolites were analyzed via a Thermo high-resolution tandem mass spectrometer in simultaneous positive/negative ion modes: parent ion scanning (70–1,050 m/z, resolution 70,000, AGC 3e6, maximum injection time 100 ms); fragment spectra were acquired in Data-Dependent Acquisition mode (resolution 17,500, AGC 1e5, maximum time 80 ms). Liquid chromatography–mass spectrometry stability was monitored via 10 quality control injections. The raw data were converted to mzXML format (via MSConvert) and processed with XCMS (peak separation/QC) and collection of algorithms for metabolite profile annotation (adduct annotation). Preliminary identification (via primary mass spectrometry and standard library matching) was performed via MetaX software, with metabolites annotated against the Human Metabolome Database (HMDB)/Kyoto Encyclopedia of Genes and Genomes (KEGG). MetaX also quantified and screened differentially abundant metabolites.

### 2.9 Statistical analysis

All the experiments were repeated three times or more, and the results are expressed as the means ± SDs. Statistical comparisons were performed for different groups via one-way analysis of variance, and the results between groups were compared via *t* tests. GraphPad Prism 9.0 software was used for data analysis and statistical charting. The difference was statistically significant at *p* < 0.05 (*p < 0.05; **p < 0.01; ***p < 0.001; ****p < 0.0001).

## 3 Results

### 3.1 Exercise intervention inhibits the progression of DMH-induced colorectal cancer

**Figure 2:**
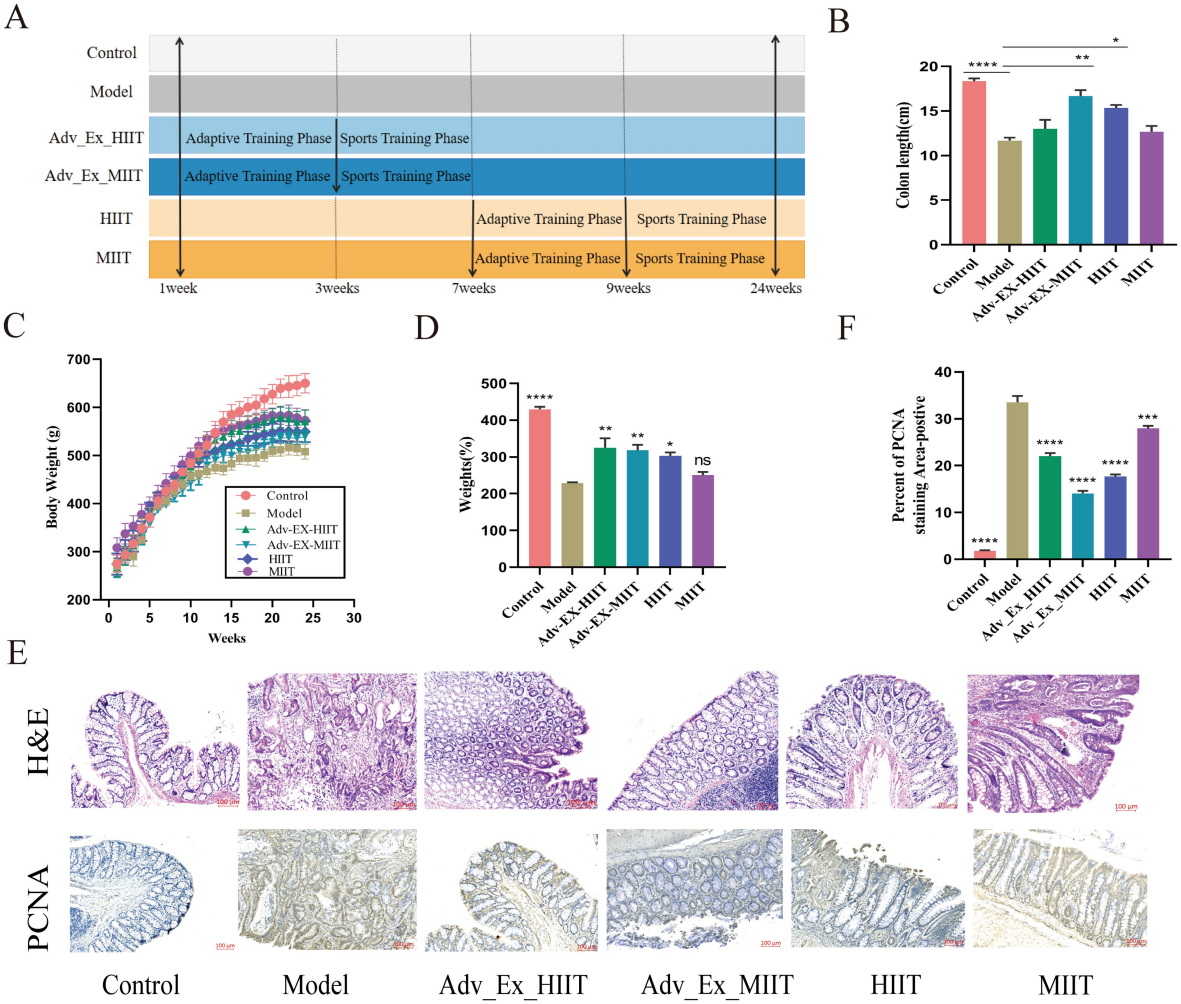
Different intensities of exercise inhibit the progression of DMH-induced colon cancer. **(A)** Schematic diagram of the experimental design. **(B)** Colon length (*n* = 6). **(C,D)** Body weight (*n* = 6). **(E,F)** Representative HE-stained sections of the colon (scale bar, 100 μm). The data are presented as the means ± SEMs. PCNA staining of colonic tissues from the indicated genotypes (scale bar: 100 μm). (*p < 0.05; **p < 0.01; ***p < 0.001; ****p < 0.0001).

To explore the role of aerobic exercise in CRC progression, we investigated its effect on the development of CRC by establishing a DMH-induced colorectal tumorigenesis model and performing exercise interventions of varying intensities (Figure 2A). Colon length reflects colorectal cancer severity: the colon length in the Model group was significantly shorter than that in the Control group, whereas Adv-Ex-MIIT and HIIT significantly alleviated this shortening (Figure 2B). During the 24-week intervention, the weekly weight data revealed that the exercise groups had significantly greater weights than did the Model group (lower than the Control group), with no significant difference between the Model and MIIT groups; varying-intensity exercise improved cancer-induced slow weight gain in the SD rats (Figures 2C,D). H&E staining revealed severe intestinal damage in the Model group (reduced goblet cells, inflammatory infiltration, etc.), with more adenocarcinoma and dysplasia, which were significantly alleviated by exercise (Figure 2E).

The Model group had more PCNA-positive cells, which were significantly reduced after exercise (Figures 2E,F). In summary, exercise of varying intensities can inhibit colorectal cancer progression.

### 3.2 Effects of exercise intervention on the intestinal flora in a rat model of colorectal cancer

**Figure 3:**
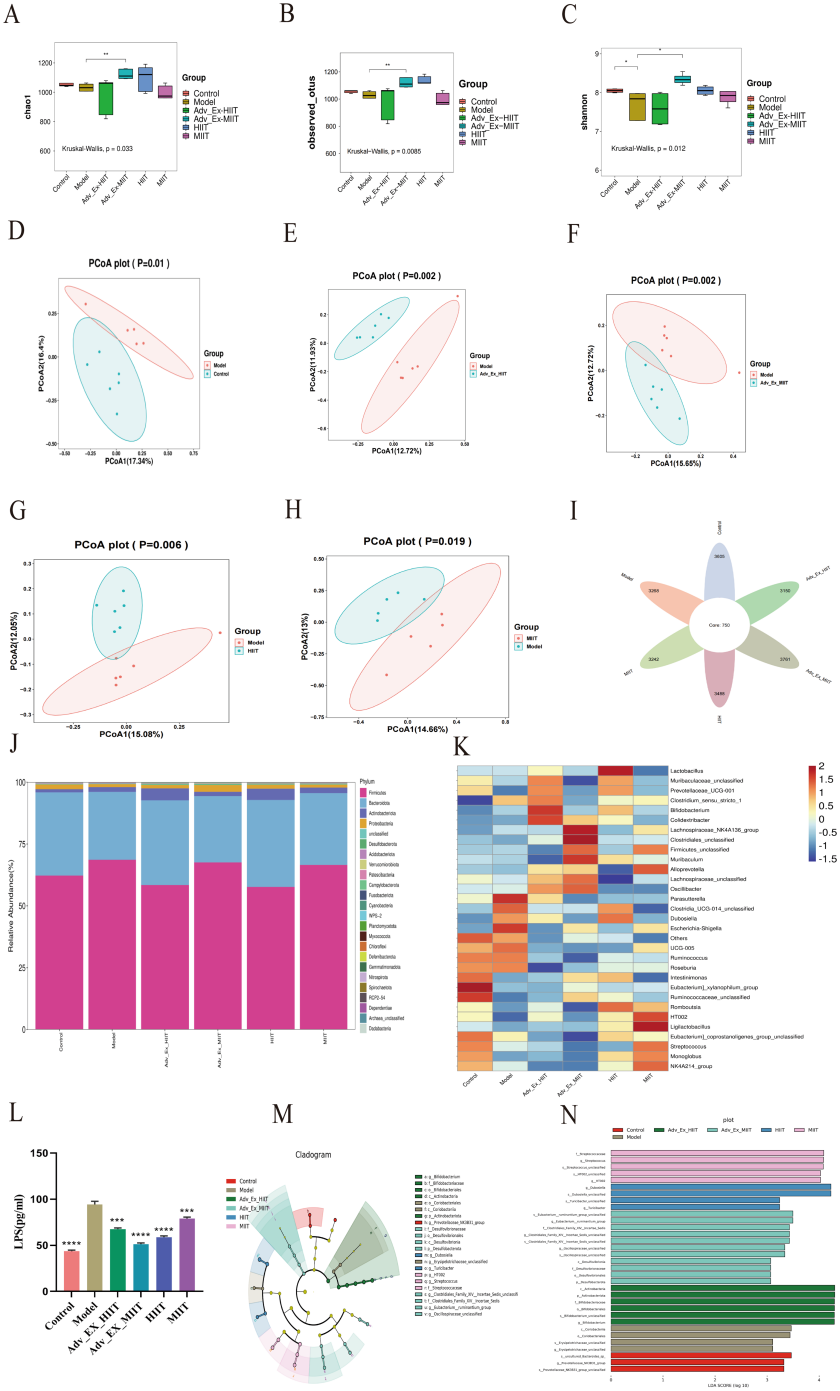
Effects of exercise intervention on the intestinal flora in a rat model of colorectal cancer. **(A)** Chao1. **(B)** Observed-otus. **(C)** Shannon index (*n* = 6). **(D–H)** PCoA. **(I)** Venn diagram of ASVs from each group (*n* = 6). **(J)** Species composition at the microbial phylum level in each group (*n* = 6). **(K)** Heatmap of microorganisms at the genus level (*n* = 6). **(L)** Concentrations of LPS in each group (*n* = 3). **(M)** LEfSe evolutionary bifurcation diagram (*n* = 6). **(N)** LDA score histogram (*n* = 6). (*p < 0.05; **p < 0.01; ***p < 0.001).

Alpha diversity characterizes species diversity, richness. There were differences in the Chao1, observed ASVs, and Shannon index of the intestinal microbiota among the different groups of rats, indicating that exercise interventions of different intensities can alter their richness and diversity. Among them, there were significant differences between the Model group and the Adv-Ex-MIIT group, suggesting that this intervention can significantly increase the richness and diversity of the intestinal microbiota in rats (Figures 3A–C).

β diversity refers to the degree of dissimilarity in species composition between different microbial communities. Compared with the control group, the model group was separated along the PCoA1 and PCoA2, indicating significant differences in intestinal microbial species between the two groups (Figure 3D). After exercise interventions of different intensities, the model group and each intervention group presented significant differences in the gut microbiota (Figures 3E–H). Exercise of different intensities can alter the colonization ability of the gut microbiota, affecting the progression of colorectal cancer to a certain extent.

Total amplicon sequence variants (ASVs) in the control, model, Adv-Ex-HIIT, Adv-Ex-MIIT, HIIT, and MIIT groups were 3606, 3268, 3150, 3761, 3488, and 3242, respectively. The intestinal bacterial richness in the Model, Adv-Ex-HIIT, and MIIT groups was lower than that in the Control, Adv-Ex-MIIT, and HIIT groups (Figure 3I). Analysis combined with the Greengenes database revealed that at the phylum level, *Firmicutes* and *Bacteroidetes* were the core flora, followed by *Actinobacteria* and *Proteobacteria*, with differences in the relative abundance of each phylum among the groups (Figure 3J).

An analysis of the gut microbiota at the genus level in rats revealed that the control group was dominated by beneficial bacteria such as *Ruminococcaceae-unclassified*, *Eubacterium j-xylanophilum group*, and *Intestinimonas*, whereas these taxa were not enriched in the model group. Following exercise interventions of varying intensities, the enrichment of these beneficial bacteria increased in the Adv-Ex-HIIT and MIIT groups. Conversely, the model group was dominated by harmful bacteria, including *Parasutterella and Escherichia-Shigella*. In the Adv-Ex-HIIT group, *Colidextribacter*, *Bifidobacterium*, *Clostridium sensu stricto-1*, and *Prevotellaceae-UCG-001* were identified as the dominant genera. For the Adv-Ex-MIIT group, *Lachnospiraceae-NK4A131, Clostridiales-unclassified,* and *Muribaculum* were the dominant genera. In the HIIT group, *Lactobacillus, Clostridium-UCG-014, Dubosiella,* and *Romboutsia* were the dominant genera. Finally, *HTO02 (unclassified genus)* and *Ligilactobacillus* were the dominant genera in the MIIT group (Figure 3K). In the model group, the increased enrichment of gram-negative bacteria, such as *Parasutterella* and *Escherichia-Shigella*, promoted lipopolysaccharide (LPS) secretion, affecting colonic cell permeability and inflammation. Compared with the control group, the model group presented higher LPS levels, which were reduced after exercise intervention (Figure 3L). In summary, exercise at varying intensities altered the colonization capacity of specific gut microbiota, enriching beneficial bacteria compared with the model group, modulating LPS levels, and inhibiting the development of colorectal cancer.

The primary objective of linear discriminant analysis effect size (LEfSe) is to identify species that exhibit significant multidimensional differences among various groups. The results of the LEfSe cladogram indicate that, at the genus level, the significantly different taxa in the fecal samples of the rats in the control group were *g-prevotellaceae-NK3B31-group (Prevotella genus)*, those in the model group were *g-Eryslpelatrichaceae-unclassified (Erysipelotrichaceae genus)*, those in the Adv-Ex-HIIT group were *C-actinobacteria (Actinobacteria genus)*, those in the Adv-Ex-MIIT group were *g-Eubacterium-ruminantium-group (Eubacterium genus)* and *g-Oscillospiraceae-unclassified (Oscillospiraceae genus),* those in the HIIT group were *g-Duboslella (Dubosiella genus)* and *g-Turicibacter*, and those in the MIIT group were *g-HTO02* and *g-Strepococcus (Streptococcus genus)* (Figure 3M). LEfSe distribution histogram analysis revealed that different intensity exercise interventions were associated with distinct microbial species. The representative microbial communities unique to each group may thus serve as potential candidates for effectively preventing colorectal cancer in rats (Figure 3N).

### 3.3 Exercise intervention can prevent the loss of intestinal barrier integrity

**Figure 4:**
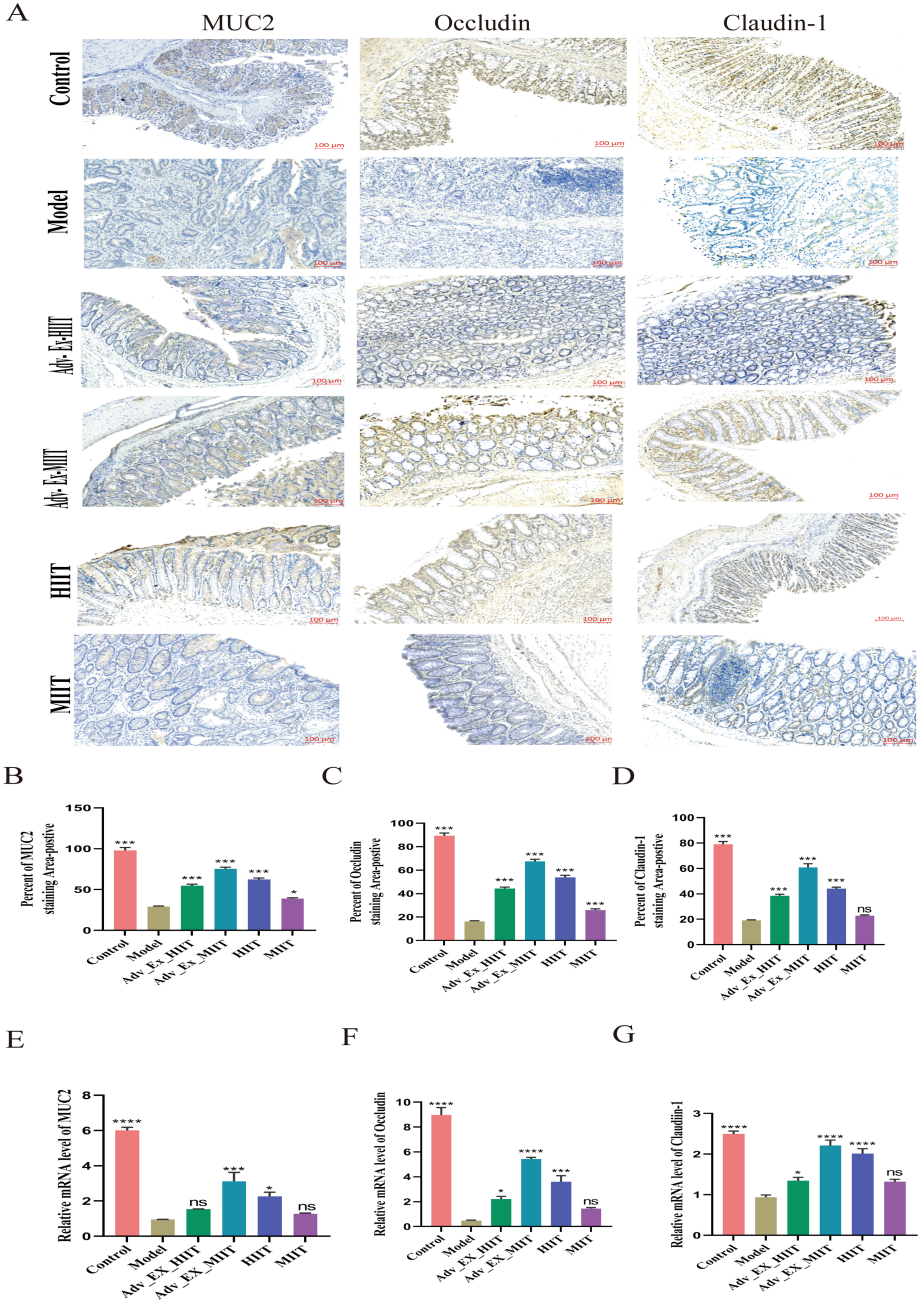
Different intensities of exercise prevent the loss of intestinal barrier integrity in rats subjected to DMH. **(A)** Representative images of immunohistochemical staining for MUC2, Occludin, and Claudin-1 in colon samples from different experimental groups (scale bar, 100 μm) (*n* = 3). **(B–D)** Statistical chart of immunohistochemical indicators. **(E–G)** Relative mRNA levels of MUC2, Occludin, and Claudin-1 (*n* = 3). (*p < 0.05; **p < 0.01; ***p < 0.001; ****p < 0.0001).

To evaluate the effects of exercise intervention on the gut microbiota and the intestinal barrier, immunohistochemistry was used to detect the expression of the tight junction proteins Claudin-1 and Occludin, as well as mucin 2 (MUC2) secreted by intestinal goblet cells. The results revealed that the protein expression levels of Claudin-1, Occludin, and MUC2 were significantly lower in the DMH rat model group than in the control group. Exercise interventions of varying intensities significantly restored intestinal barrier integrity, with notable differences observed among the groups. However, no significant difference in Claudin-1 protein expression was found between the model and MIIT groups (Figure 4A). Statistical charts of the immunohistochemical indices are presented in Figures 4B–D. Subsequent qRT–PCR analysis of claudin-1, occludin, and MUC2 mRNA levels revealed protein expression trends; however, no significant differences in these markers were detected between the model and MIIT groups (Figures 4E–G).

### 3.4 Effects of exercise intervention on intestinal inflammation in rats with DMH-induced colorectal cancer

**Figure 5:**
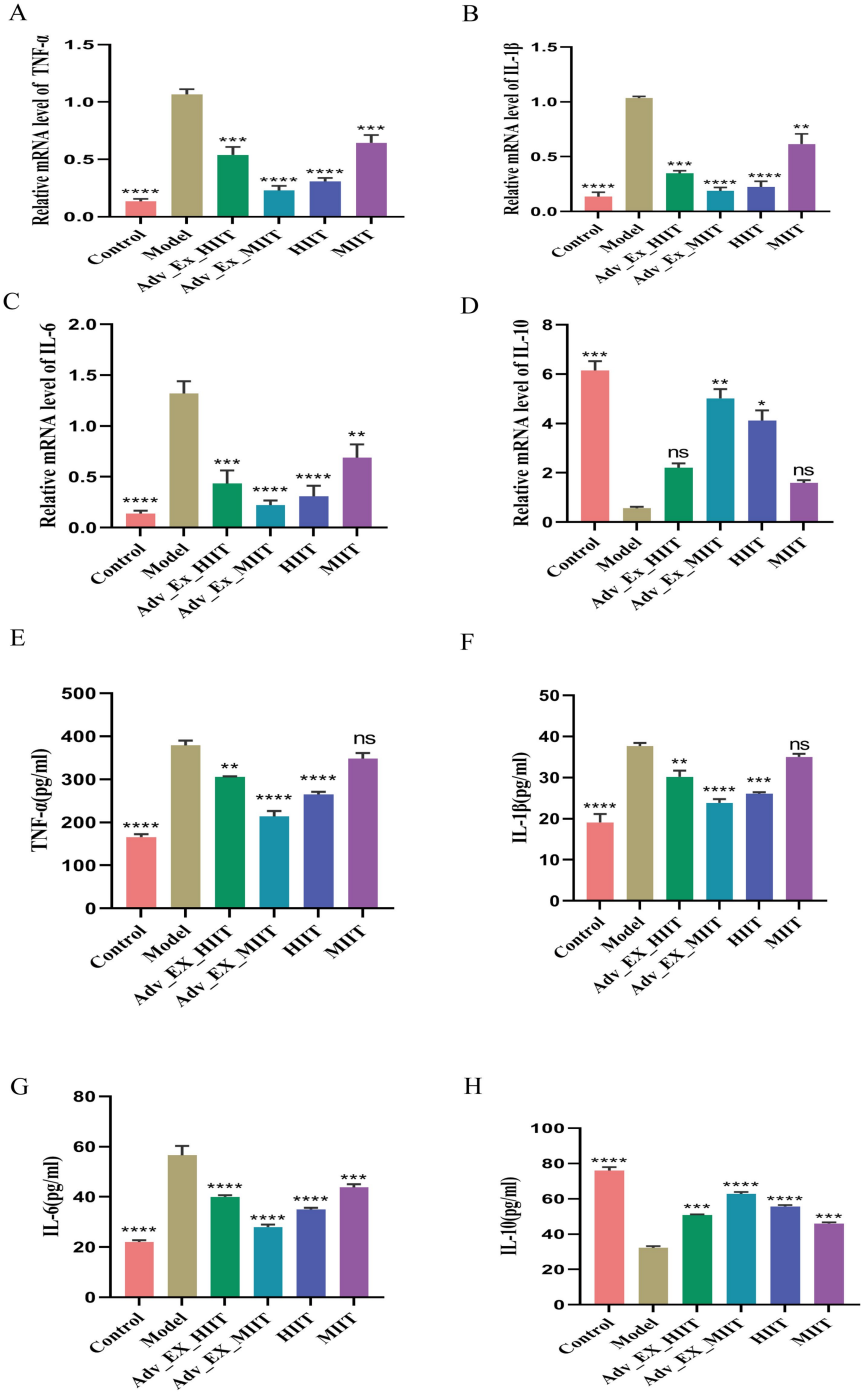
Effects of exercise of different intensities on intestinal inflammation in DMH-induced colon cancer rats. **(A–D)** Expression of the inflammatory factors IL-1β, TNF-α, IL-6, and IL-10 in colon tissue (*n* = 3). **(E–H)** Supernatants. Relative mRNA expression of inflammatory factors in the colon was evaluated by qRT–PCR (*n* = 3) (*p < 0.05; **p < 0.01; ***p < 0.001; ****p < 0.0001).

qRT–PCR and ELISA were used to assess the impact of gut microbiota regulation on inflammatory factors following exercise interventions of varying intensities. The results revealed that the levels of proinflammatory cytokines (IL-1β, TNF-α, and IL-6) were significantly elevated and that the level of the anti-inflammatory cytokine IL-10 was reduced in the model group, whereas these trends were reversed after exercise. No significant differences in the levels of IL-10 or certain proinflammatory cytokines were observed between the model and MIIT groups (Figures 5A–H).

### 3.5 Effects of exercise intervention on intestinal Flora-biochemical index correlations and fecal metabolites in a rat model of colorectal Cancer

**Figure 6:**
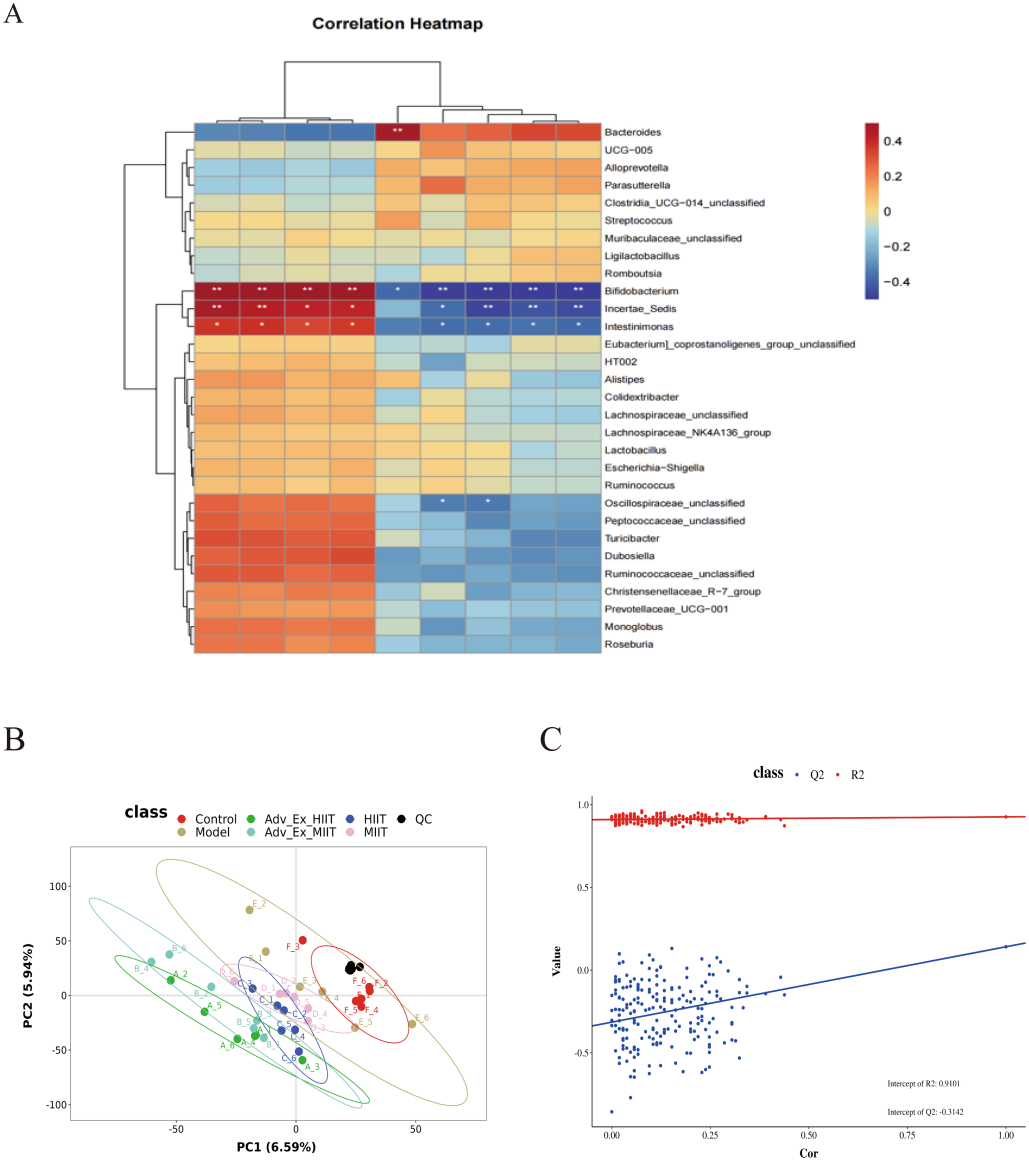
Influences of exercise intervention on intestinal flora–biochemical index correlations and fecal metabolites in a rat model of colorectal cancer. **(A)** Correlation analysis between the intestinal microbiota and biochemical indices. **(B)** PLS-DA scores of the fecal metabolites in the rats in each group. **(C)** RPT test of the PLS model of fecal metabolites in the rats in each group. (*p < 0.05; **p < 0.01; ***p < 0.001).

Further exploration of the correlations between the biochemical indicators after aerobic exercise interventions of different intensities and the top 30 intestinal microbiota at the genus level with significant differences was conducted. The results revealed significant differences in the associations with Bifidobacterium, Incertae-Sedis, Bacteroides, Intestinimonas, and Oscillospiraceae. Among them, MUC2, Occladin, Claudin-1, and IL-10 were positively correlated with Bifidobacterium, Incertae-Sedis, and Intestinimonas and negatively correlated with Bacteroides. In addition, IL-6, IL-1β, TNF-α, PCNA, and LPS were negatively correlated with *Incertae-Sedis, Bifidobacterium, Intestinimonas,* and *Oscillospiraceae unclassified* and *positively correlated with Bacteroides*. These findings indicate that exercise intervention protects the integrity of the intestinal barrier and regulates the immune response to inhibit the progression of colorectal cancer by modulating the balance of the intestinal microbiota in rats (Figure 6A).

Rat feces were subjected to nontargeted detection of metabolites (LC–MS/MS) via a high-resolution tandem mass spectrometer, Q Exactive (Thermo Scientific). The collected metabolites were subsequently compared and intersected with the HMDB and KEGG databases, and a total of 1,114 fecal metabolites were identified. The results revealed that there were differences in the metabolites in each group. The Model group is similar to the MIIT group, and the Adv-Ex-HIIT group is similar to the Adv-Ex-MIIT group (Figure 6B). The permutation test graph shows the linear regression to measure whether the model is overfitted. When R2 > 0.5 is close to 1, it is better. The starting point of Q2 on the longitudinal axis is <0, indicating that there is no overfitting phenomenon in the model and that the analysis of differentially abundant metabolites is more accurate (Figure 6C).

### 3.6 Comparative analysis of fecal differentially abundant metabolites, annotation and enrichment of their pathways, and correlation with microbial communities

**Figure 7:**
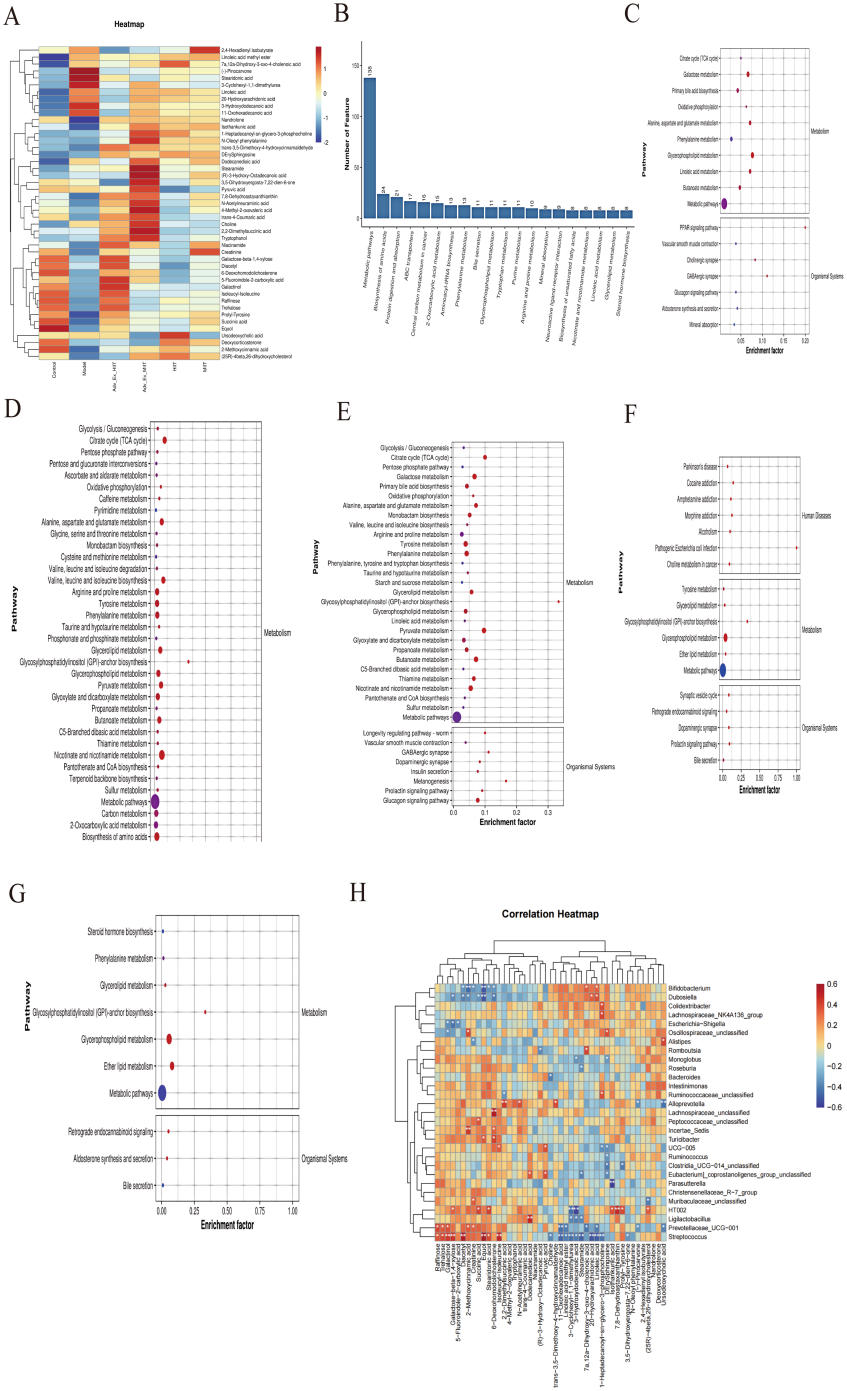
Comparative analysis of fecal differential metabolites, annotation, and enrichment of their pathways, and correlation with microbial communities. **(A)** Comparative analysis of different fecal metabolites between groups. **(B)** Top 20 metabolic pathways. **(C)** KEGG enrichment analysis of metabolic pathways in the model and control groups. **(D)** KEGG enrichment analysis of metabolic pathways in the Adv-Ex-HIIT and model groups. **(E)** KEGG enrichment analysis of metabolic pathways in the Adv-Ex-MIIT and model groups. **(F)** KEGG enrichment analysis of metabolic pathways in the HIIT and model groups. **(G)** KEGG enrichment analysis of metabolic pathways in the MIIT and model groups (*n* = 6). **(H)** Correlation analysis between microbial communities and metabolites (*n* = 6) (*p < 0.05; **p < 0.01; ***p < 0.001).

To further investigate the effects of different-intensity exercise interventions on intestinal metabolites, we screened differentially abundant metabolites on the basis of a VIP value >1 and a *t* test *p* value <0.05 and visualized them via heatmaps, selecting 46 such metabolites to analyze their expression patterns across groups. Compared with the Control group, the Model group presented significant upregulation of lipid metabolites associated with gut microbiota metabolism, including *stearic acid, methyl linoleate, 20-hydroxyeicosatetraenoic acid, 3-oxodeoxycholic acid,* and *3-hydroxydodecanoic acid*; these lipids, which were elevated in the Model group, were significantly downregulated in the Control group, and their enrichment was reduced following different-intensity exercise interventions. In contrast, the control group presented significantly greater levels of organic oxygen compounds related to gut microbiota metabolism, such as raffinose, β-galactosyl-(1 → 4)-xylose, trehalose, 3-oxodeoxycholic acid, and succinic acid, than did the model group, with exercise interventions at different intensities partially restoring the enrichment of these compounds. Additionally, each exercise intervention group displayed unique enrichment of specific metabolites beyond these shared changes, highlighting the distinct metabolic profiles induced by varying exercise intensities (Figure 7A).

The significantly different metabolites were analyzed for KEGG enrichment, and the top 20 pathways with significant enrichment were selected from the pathways (Figure 7B). The main differential metabolic pathways in the Model and Control groups were significantly enriched in amino acid metabolism, bile acid metabolism, and lipid metabolism, including aspartic acid, alanine, and glutamate metabolism; phenylalanine and butyric acid metabolism; linoleic acid metabolism; glycerol phospholipid metabolism; the tri**c**arboxylic acid cycle (TCA cycle); and primary bile acid metabolism (Figure 7C). The changes in metabolites in the feces of the rats in the Adv-Ex-HIIT group and Model group were associated mainly with fatty acid metabolism pathways, bile acid metabolism pathways, and amino acid and glycerol–phospholipid metabolism pathways, among which the significant ones were butyric acid, propionic acid, pyruvate, primary bile acid and tyrosine, and glycerol–phospholipid metabolism pathways (Figure 7D). Compared with those of the Adv-Ex-MIIT group and Model group, the differential metabolic pathways of the rats were not enriched with linoleic acid and primary bile acid metabolism pathways, whereas carbohydrate, phenylalanine, arginine, proline, isoleucine and 2-oxycarboxylic acid metabolism pathways were enriched (Figure 7E). The metabolic pathways of the MIIT and Model groups were similar to those of the HIIT and Model groups and were enriched mainly in glycerol phospholipid metabolism (Figures 7F,G). In summary, exercise interventions of different intensities can affect multiple pathways and thus affect the progression of colorectal cancer.

The correlations between the differentially abundant metabolites and the microbial community structure (top 30 at the genus level) were further analyzed via Spearman correlation analysis. The results revealed that Bifidobacterium (Bifidobacterium) and Dubosiell (Ducai) were positively correlated with the metabolites 3-oxodeoxycholic acid, 20-hydroxyeicosantetraenoic acid, and linoleic acid (linoleic acid) and negatively correlated with 2,3-butanedione, 2-methoxycinnamic acid, and inosine. Eubacteriumj-coprostanoligenes-group-unclassified was negatively correlated with stearamide and positively correlated with deoxycholic acid and pyruvate, a primary fatty acid amide with cytotoxicity. *Streptococcus* is negatively correlated with linoleic acid, and studies have also shown that linoleic acid is beneficial to the body. *Clostridia-UCG-014 (Clostridia)* was negatively correlated with stearamide and Prolyl-Tyrosine. *Escherichia-Shigella (Egyptian-Shigella)* is negatively correlated with betulinic acid and 5-fluorindole-2-carboxylic acid. The above results show that different intensities of aerobic exercise significantly improve bile acid and lipid metabolism and that aerobic exercise intervention can influence the progression of colorectal cancer by regulating the interaction between intestinal microorganisms and their metabolites (Figure 7H).

## 4 Discussion

Currently, colorectal cancer remains a difficult-to-diagnose and fatal cancer. It is a multifactorial disease closely linked to factors such as low physical activity, obesity, high BMI, a high-fat low-fiber diet, alcohol consumption, smoking, and family history (Neumann et al., 2015). Many studies have shown that regular moderate-to-intense physical activity reduces the risk of many cancers, delays cancer development, and improves patient prognosis and quality of life (Wang et al., 2022).

In this study, colorectal cancer rats were subjected to different intensities of exercise. After 6 months, Adv-Ex-MIIT and HIIT significantly improved DMH-induced colon shortening, slowed weight gain, and inhibited cell proliferation. To a certain extent, the damage to the colonic structure was reversed, which is consistent with previous research results (Ren et al., 2021).

Studies have shown that exercise can individually affect the structure and function of the intestinal microbiota. Compared with sedentary hypercholesterolemic mice, hypercholesterolemic mice subjected to a 12-week running wheel presented increased bile acid secretion and increased fecal bile acid output (Meissner et al., 2011). Strong and high-intensity exercise can also have a significant effect on the intestinal barrier, which rapidly renews cells, and high energy demands make the structure fragile, thereby increasing the risk of colorectal cancer (Bäumler and Sperandio, 2016). In mouse models, strenuous exercise promotes intestinal inflammation and increases the growth of *Butivirella*, *cyclic spirochetes*, and *Coprococcus* while reducing the *Turicibacter* (Clark and Mach, 2022). Currently, few studies on exercise interventions of different intensities that regulate the intestinal microbiota to affect the progression of colorectal cancer exist; thus, we explored this topic by detecting 16S rRNA and nontargeted metabolites. The results showed that exercise intervention could alter intestinal species richness and community diversity in DMH chemically induced colorectal cancer rats, among which the differences in the Adv-Ex-MIIT group were significant.

Many colorectal cancer studies have shown that the level of SCFAs in the feces of CRC patients has decreased, which can be attributed to the decrease in the number of microorganisms that produce SCFAs, such as *Bifidobacterium* and *Lachnospira,* which in turn promotes the progression of colorectal cancer (Wang et al., 2011). According to our study, *Bifidobacterium*, *Clostridium sensu stricto-1*, *Prevotella-UCG-001*, and *Clostridia* (*Escherichia coli*) are the dominant microbiomes of the Adv-Ex-HIIT group. *Prevotellae-UCG-001* are common beneficial bacteria, and coliform bacteria (*Escherichia coli*) are common harmful bacteria in the intestines. This finding shows that harmful bacteria and beneficial bacteria are regulated simultaneously under the intervention of the Adv-Ex-HIIT group, which leads to a lack of significant effects on colorectal cancer compared with those of the Adv-Ex-MIIT group. *Lachnospiraceae-NK4A131 (Lucidialis), Clostridiales-unclassified,* and *Muribaculum (Epimorius)* were the dominant microbial groups of the Adv-Ex-MIIT group. Studies have shown that Firmicutes account for the greatest proportion of bacteria in the intestine, accounting for more than 60% of bacteria. *Lactobacillus*, which is a typical probiotic, can maintain the acidic internal environment of the intestine (Arumugam et al., 2011). Studies have also shown that supplementation and reduced symbionts are effective in improving intestinal infections. For example, inflammation caused by *C. difficile* is improved by colonization with *C. difficile* (Buffie et al., 2014). *Lactobacillus, Clostridia-UCG-014, Dubosiella,* and *Romboutsia* were the dominant microorganisms in the HIIT group. *Saliva* is a lactic acid bacteria that has attracted attention as a promising probiotic, many of which exhibit functional properties with health benefits such as antibacterial activity, immune effects, and the ability to regulate the gut microbiota (Zhou et al., 2022). A decrease in the level of LIG in tobacco (*Lactobacillus salivary*) can aggravate the development of colon inflammation. According to our research, *HTO02* and *Listeria (Lactobacillus salivary)* are the dominant microbial groups of the MIIT group. The harmful bacteria *Escherichia-Shigella* and *Streptococcus* were the dominant microbial groups in the Model group. The above results show that exercise intervention may inhibit the progression of colorectal cancer by increasing the abundance of *Bifidobacterium, Latex, Clostridium, Lactobacillus,* and *Lactobacillus salivary liposula* and reducing the abundance of *Espionia-Shigella* and *Streptococcus*. According to previous studies, Bifidobacterium can protect the intestinal barrier (Arumugam et al., 2011). The increased enrichment of two gram-negative bacteria, *Parasutterella (Parasalmonella)* and *Escherichia-Shigella (Egyptia-Shigella),* promotes the secretion of LPS and thus affects the permeability and inflammation level of colon cells. In this study, exercise interventions of different intensities were used to protect the intestinal barrier integrity of rats at the protein level, and there was no significant difference in the MIIT group at the mRNA level. In addition, epidemiological studies have shown that exercise is related to lower circulating levels of TNF**-**α, IL-6 and CRP. Compared with a sedentary lifestyle, physical activity can reduce the level of peripheral inflammatory mediators by 20%–60% (Zhou et al., 2022), which is consistent with our research results, indicating that exercise interventions of different intensities can effectively inhibit the release of proinflammatory factors and promote the expression of anti-inflammatory factors, thereby inhibiting the progression of colorectal cancer.

To clarify the correlations between specific microbiota in the intestine and inflammatory factors and the intestinal barrier, we conducted correlation analysis and found significant correlations with *unclassified Bifidobacterium, Incertae-Sedis, Bacteroides, Intestinimonas,* and *Oscillospiraceae.* In this study, metabolites such as amino acids, bile acids, and fatty acids were detected in the rat intestinal flora through nontargeted metabolomic analysis. The Adv-EX-HIIT and Adv-EX-MIIT groups affected the progression of colon cancer by affecting butyric acid metabolism, primary bile acid metabolism, and glycerol phospholipid metabolism; the HIIT and MIIT groups affected mainly glycerol phospholipid metabolism pathways. The main metabolites involved are linoleic acid, 3-oxodeoxycholic acid, 20-hydroxyeicosantetraenoic acid, 2,3-butanedione, 2-methoxycinnamic acid, stearamide, deoxycholic acid, and pyruvate. Finally, we further analyzed the correlation between metabolites and the intestinal flora and found that Eubacteriumj-coprostanoligenes-group-unclassified metabolites were negatively correlated with stearamide and positively correlated with deoxycholic acid and pyruvate. Stearamide is a primary fatty acid amide. According to previous studies, stearamide is cytotoxic. *Bifidobacterium (Bifidobacterium) and Dubosiell (Ducai)* were positively correlated with the metabolites 3-oxodeoxycholic acid, 20-hydroxyeicosantetraenoic acid, and linoleic acid and negatively correlated with 2,3-butanedione, 2-methoxycinnamic acid, and inosine C.

This study explored exercise-induced remodeling of the gut microbiota in rats with colorectal cancer. Different exercise intensities had varying effects on gut microbial colonization, but all inhibited cancer progression—with the Adv-EX-HIIT and HIIT groups showing the most significant effects. The mechanism involves exercise-induced remodeling of the microbiota to protect the intestinal barrier and regulate inflammation. However the current study has several limitations. First, the dimethylhydrazine (DMH)-induced carcinogenesis model does not fully recapitulate the complexity of human colorectal cancer. Second, the exclusive use of male rats precludes the assessment of sex-based differences. Furthermore, a key limitation is the lack of mechanistic validation (e.g., fecal microbiota transplantation, antibiotic treatment, metabolite supplementation) to confirm causal links between gut microbiota, metabolites, inflammatory factors, and CRC progression.

## 5 Conclusion

Exercise intervention can inhibit the proliferation of colorectal cancer cells.

Exercise intervention can reshape the intestinal microbiota and its metabolites in a rat colorectal cancer model, increase the expression of beneficial bacteria, and reduce the expression of harmful bacteria.

The intestinal microbiota and its metabolites remodeled by exercise intervention can partially restore the structural function of the intestinal barrier, regulate the level of inflammatory factors, and further inhibit the progression of colon cancer.

## Data Availability

The data presented in this study are publicly available. The data can be found here: https://www.ncbi.nlm.nih.gov/sra,PRJNA1406033.
