# Correction: Gut microbiota affects the progression of colorectal cancer under the intervention of exercise

**DOI:** 10.3389/fmicb.2026.1833893

**Published:** 2026-04-20

**Authors:** Linlin Tao, Huan Zhou, Wenjiao Shao, Dongmei Liu, Yingwen Ruan, Mingwei Chen

**Affiliations:** 1 Department of Human Anatomy, Harbin Medical University, Harbin, China; 2 Department of Radiology, Renmin Hospital of Wuhan University, Wuhan, China; 3 Department of Gynecological Radiotherapy, Harbin Medical University Cancer Hospital, Harbin, China

**Keywords:** colorectal cancer, exercise intervention, gut microbiota, inflammation, intestinal barrier

In the published article, there was an error in the affiliations for authors Linlin Tao, Huan Zhou, and Wenjiao Shao. Affiliations 1 and 2 were erroneously swapped and affiliation 2 was omitted for Linlin Tao.

The correct author and affiliation list appears below:

Linlin Tao^1, 2^, Huan Zhou^1^, Wenjiao Shao^1^, Dongmei Liu^3^, Yingwen Ruan^3^ and Mingwei Chen^1*^

^1^ Department of Human Anatomy, Harbin Medical University, Harbin, China

^2^ Department of Radiology, Renmin Hospital of Wuhan University, Wuhan, China

^3^ Department of Gynecological Radiotherapy, Harbin Medical University Cancer Hospital, Harbin, China

The original version of this article has been updated.

