# Supplementary material for: Gut microbiota affects the progression of colorectal cancer under the intervention of exercise
Source: Front Microbiol. 2026 Feb 5;17:1728541. doi: 10.3389/fmicb.2026.1728541 (PMC12916588; doi:10.3389/fmicb.2026.1728541)
Supplement: Supplementary file 1 [file Data_Sheet_1.pdf]

Supplementary information

Gut microbiota affects the progression of colorectal cancer under the intervention of exercise

Linlin Tao[1,#], Huan Zhou[2,#], Wenjiao Shao[2], Dongmei Liu[3], Yingwen Ruan[3], Mingwei Chen[1,*]

(1. Department of Radiology, Renmin Hospital of Wuhan University, 238 Jiefang Road, Wuchang District, Wuhan 430060, China; 2. Department of Anatomy, Harbin Medical University, No. 157 Baojian Road, Nangang District, Harbin 150081, China; 3. Department of Gynecological Radiotherapy, Harbin Medical University Cancer Hospital, No. 150 Haping Road, Nangang District, Harbin 150081, China; # Contributed equally; * Corresponding Author)

Supplementary Figures

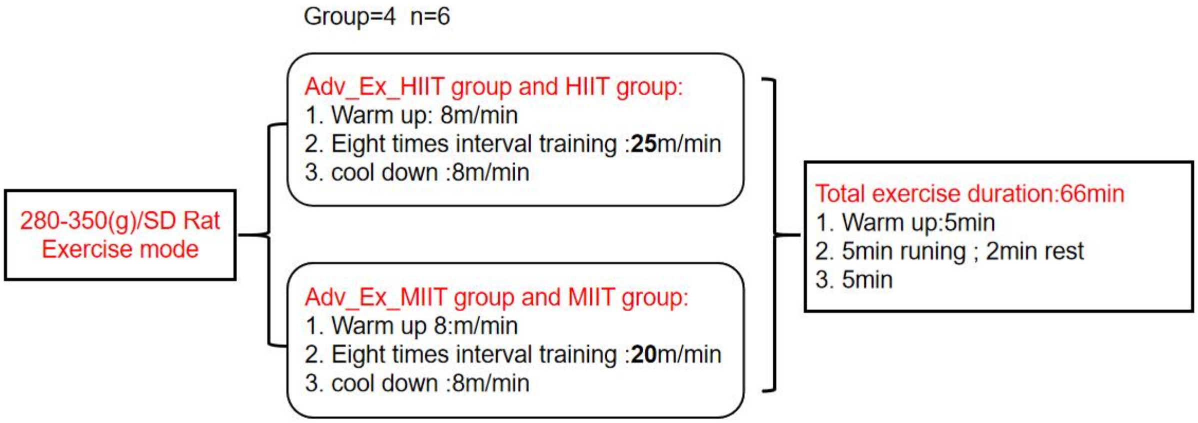

Supplementary Fig. S1 **Motion pattern diagram**

Supplementary tables

Occludin	Wanleibio
Claudin 1	Wanleibio
PCNA	Proteintech
MUC2	Affinity
Goat Anti-Rabbit IgG (H+L) antibody	Affinity

Supplementary Table S1. List of antibodies used this study.

Murine gene	Forward（5’-3’）	Reverse(5’-3’)
GAPDH	TGATGGCAACAATGTCCACT	AGACAGCCGCATCTTCTTGT
IL-6	TGGCTGGAAGTCTCTTGCGG	GCCCACCAGGAACGAAAGTC
IL-1β	AGTCAAGGGCTTGGAAGCAA	GGCTTCCTTGTGCAAGTGTC
TNF-α	GCTTGGTGGTTTGCTACGAC	ATGGGCTCCCTCTCATCAGT
IL-10	GACACCTTTGTCTTGGAGCTTA	TTCCCTGGGAGAGAAGCTGA
Claudin1	CACTAATGTCGCCAGACCTGA	TGGGGCTGATCGCAATCTTT
Occludin	GCCTGTAAGGAGGTGGACTC	GGGGCGCAGCAGGTCT
MUC2	GTACCGCAGGGGACATTCTC	TGTGGCTGTCCAGGACTACT
PCNA	TCATCTTCGATCTTGGGAGCC	CTGCAGATGTACCCCTTGTTGT

Supplementary Table S2. qRT-PCR primers used in this study.

Ingredients	Reaction system
RNA	1μg
5×RT Buffer	4μl
Primer Mix	1μl
RT Enzyme Mix	1μl
Nuclease-free Water	Up to 20μl

Supplementary Table S3. Reverse transcription reaction system

Ingredients	Reaction system
ddH2O	7.2μl
SYBR Green	10μl
Forward primer	0.4μl
Reverse primer	0.4μl
cDNA	2μl

Supplementary Table S4. qRT-PCR Reaction system

Stage1	95℃	30s
Stage2	95℃	5s
	50-60℃	30s
	72℃	60s
	Total 50 cycle	

Supplementary Table S5. qRT-PCR Reaction procedure

Kit Name	source
Rat IL-1β ELISA detection kit	Shanghai Jingkang Biotechnology Co., Ltd.
Rat TNF-α ELISA detection kit	Shanghai Jingkang Biotechnology Co., Ltd.
Rat IL-6 ELISA detection kit	Shanghai Jingkang Biotechnology Co., Ltd.
Rat IL-10 ELISA detection kit	Shanghai Jingkang Biotechnology Co., Ltd.
Rat LPS ELISA detection kit	Shanghai Jingkang Biotechnology Co., Ltd.

Supplementary Table S6. Kits are used in ELISA experiments

Ingredients	PCR reaction system
Phusion Hot start flex 2X Master M	12.5 µl
Forward Primer	2.5 µl
Reverse Primer	2.5 µl
Template DNA	50 ng
Add ddH2O to	25 µl

Supplementary Table S7. PCR reaction system for 16SrRNA sequencing

Reaction temperature	Reaction time	Number of cycle
98 ℃	30s	
98 ℃	10s	
54 ℃	30s	35 cycle
72 ℃	45s	
72 ℃	10min	
4 ℃	∞	

Supplementary Table S8. PCR reaction produce for 16SrRNA sequencing

Amplified segment	nucleotide sequence
V3-V4	F(5'-CCTACGGGNGGCWGCAG-3')
	R(5'-GACTACHVGGGTATCTAATCC-3')
Archaeons	F(5'-GYGCASCAGKCGMGAAW-3')
	R(5'-GGACTACHVGGGTWTCTAAT-3')
V4	F(5'-GTGYCAGCMGCCGCGGTAA-3')
	R (5'- GGACTACHVGGGTWTCTAAT-3')
V4-V5	F(5'-GTGCCAGCMGCCGCGG-3')
	R(5'-CCGTCAATTCMTTTRAGTTT-3')

Supplementary Table S9. PCR Amplified fragments for 16SrRNA sequencing
